# Charge-order domain walls with enhanced conductivity in a layered manganite

**DOI:** 10.1038/ncomms8595

**Published:** 2015-07-03

**Authors:** Eric Yue Ma, Benjamin Bryant, Yusuke Tokunaga, Gabriel Aeppli, Yoshinori Tokura, Zhi-Xun Shen

**Affiliations:** 1 Department of Applied Physics, Stanford University, Stanford, California 94305, USA; 2 London Centre for Nanotechnology and Department of Physics and Astronomy, University College London, London WC1E 6BT, UK; 3 RIKEN Center for Emergent Matter Science (CEMS), Wako, Saitama 351-0198, Japan; 4 Department of Physics, ETH Zürich, CH-8093 Zürich, Switzerland; 5 Department of Physics, École Polytechnique Fédérale de Lausanne (EPFL), CH-1015 Lausanne, Switzerland; 6 Synchrotron and Nanotechnology Department, Paul Scherrer Institute, CH-5232, Villigen, Switzerland; 7 Department of Applied Physics, University of Tokyo, Bunkyo-ku, Tokyo 113-8656, Japan

## Abstract

Interfaces and boundaries in condensed-matter systems often have electronic properties distinct from the bulk material and thus have become a topic of both fundamental scientific interest and technological importance. Here we identify, using microwave impedance microscopy, enhanced conductivity of charge-order domain walls in the layered manganite Pr(Sr_0.1_Ca_0.9_)_2_Mn_2_O_7_. We obtain a complete mesoscopic map of surface topography, crystalline orientation and electronic phase, and visualize the thermal phase transition between two charge-ordered phases. In both phases, charge-order domains occur with domain walls showing enhanced conductivity likely due to local lifting of the charge order. Finite element analysis shows that the resolved domain walls can be as narrow as few nanometres. The domain walls are stabilized by structural twins and have a strong history dependence, suggesting that they may be manipulated to create novel devices.

## Abstract (web summary)

Interfaces can give rise to novel states of matter not found in bulk materials. Here, the authors use microwave impedance microscopy to show that the conductivity at charge-order domain walls in a layered manganite is higher than in the rest of the material, due to local lifting of the charge order.

## Graphical abstract

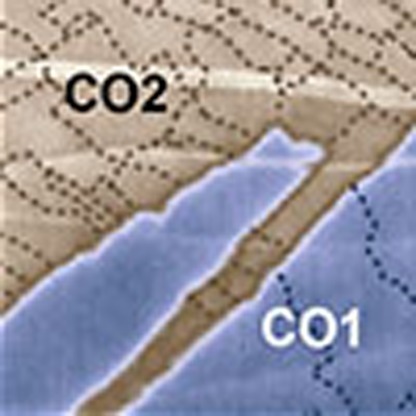

Recent advances in material growth, nano-fabrication and microscopy have enabled the study of microscopic interfaces and boundaries, which often possess properties different from the bulk material—for example, conductive interfaces between insulators^1^, superconductivity enhanced by interfacial mode coupling^2^ and multiferroic heterostructures^3^,^4^,^5^,^6^. Of significant current interest are the electrical properties of order parameter domain boundaries in chemically homogeneous systems, in particular, ferroelectric domain walls and nano-domains, where the order parameter—spontaneous polarization—changes abruptly within inter-atomic scales and the resulting bound and screening charges are believed to strongly alter the electronic conduction^7^,^8^,^9^,^10^,^11^,^12^,^13^,^14^,^15^. In this work, we show that domain boundaries of another order parameter, charge order, may also have novel electronic properties in a chemically homogeneous bulk material.

Charge-ordered (CO) phases occur in many mixed-valence manganites, where Mn^4+^ and Mn^3+^ ions are arranged in a ‘checkerboard' pattern^16^. The coexisting orbital order, magnetism, ferroelectricity and structural distortions add more degrees of freedom, creating an ideal circumstance for emergent phenomena at interfaces^17^,^18^. The half hole-doped bilayer manganite Pr(Sr_0.1_Ca_0.9_)_2_Mn_2_O_7_ (PSCMO) exhibits two distinct charge- and orbital-ordered (CO-OO) phases with a hysteretic transition near room temperature (Fig. 1a)^19^,^20^,^21^. Due to charge order superposed on the orthorhombically distorted bilayer crystal structure, the lower temperature charge-ordered phase (CO2) is ferroelectric, whereas the higher temperature phase (CO1) is layered anti-ferroelectric^22^. While optical second-harmonic generation measurements have provided evidence of domains in the ferroelectric CO2 phase^23^, the electrical properties of the domain walls remain unknown, mainly due to the difficulty of measuring local transport in a bulk crystal. Moreover, no domain structure has been identified in the anti-ferroelectric CO1 phase.

In the following, we report how we have used microwave impedance microscopy (MIM), a scanning probe technique that measures the GHz local electromagnetic response of samples without needing a back electrode to pass currents^24^,^25^, to visualize the local conductivity difference between the two CO phases and to identify additional emergent behaviours within both phases, namely charge-order domain walls that show enhanced conductivity.

## Results

### Mapping charge-order phase coexistence

**Figure 1:**
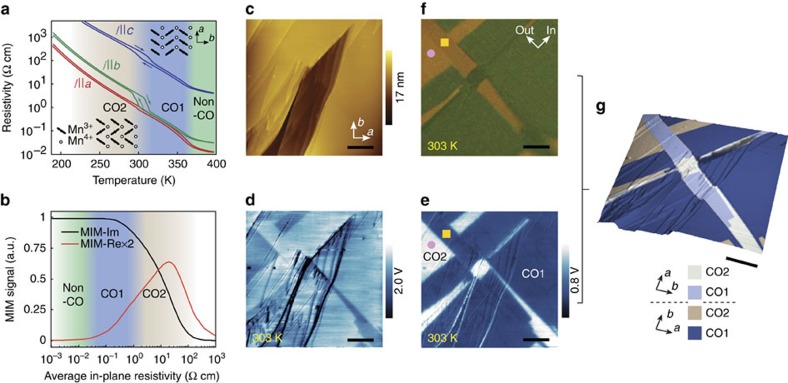
Complete mesoscopic topography/crystalline/electronic phase mapping of PSCMO. (**a**) Resistivity of PSCMO versus temperature, for in- and out-of-plane directions (reproduced from ref. 19). Insets show the CO-OO patterns. (**b**) MIM response curve obtained by simulating the complex admittance between the MIM tip and a realistic model of the material. Resistivity of the different phases in the relevant temperature range is marked. (**c**) AFM topographic image of a 25 by 25 μm twinned region at 303 K. (**d**) MIM-Im and (**e**) MIM-Re images, which correspond to the imaginary and real part of tip–sample admittance at 1 GHz, of the same area. The contrast between the two charge-ordered phases is clear. The step edges appear in MIM images because the tip–sample admittance, especially the imaginary (capacitive) part, is perturbed at sharp topographic features. (**f**) Cross-polarized light microscopy image of the same area, showing optical anisotropy contrast. The fact that the two regions marked by the pink circle and yellow square in **e** and **f** have conductivity contrast, but no optical contrast immediately tells us that they are in different phases and have crystalline directions that differ by 90°. (**g**) Surface topography overlaid with crystal orientation and charge-order phases, deduced from **c**–**f**, indicated. Scale bars, 5 μm.

We first establish the capability of MIM for the study of PSCMO by obtaining a complete mesoscopic two-dimensional map of surface topography, crystalline direction and electronic phase (Fig. 1). MIM scans were performed on a cleaved surface of a PSCMO crystal at 303 K, where coexistence of CO1 and CO2 phases is expected. Figure 1c shows the surface topography: atomically flat regions of ∼10 μm extent are separated by step edges representing single or multiple bilayers. By delivering a 1 GHz excitation of 5 μW to a metallic tip, images of the real (resistive) and imaginary (capacitive) parts of the tip–sample complex admittance during the scanning were produced, henceforth referred to as MIM-Re and MIM-Im^24^,^26^. Figure 1d,e show MIM-Im and MIM-Re images revealing clear conductivity contrasts. According to the MIM response curve obtained by finite element analysis (Fig. 1b, Supplementary Fig. 1, Supplementary Note 1), the CO1 phase is expected to have a higher signal than the CO2 phase in MIM-Im images and the opposite in MIM-Re images: therefore, we may identify the bright (dark) areas in the MIM-Re (MIM-Im) image as CO2 and the dark (bright) areas as CO1. Strikingly, the electronic phase pattern has little correlation with surface topography, confirming its bulk origin. We then use cross-polarized light microscopy (PLM) to map optical anisotropy contrast, which can have two origins in this material: 90° rotation of OO stripes between CO1 and CO2 phases within a single crystalline region (Fig. 1a)^27^, and 90° rotation of crystalline orientation between structural twins of the same phase. By comparing the PLM (Fig. 1f) and MIM-Re (Fig. 1e) image of the same area, one can easily distinguish both the difference in electronic phase and the difference of crystalline orientation due to structural twins, to construct a complete map as in Fig. 1g. It appears that the low-temperature CO2 phase is stabilised near the twin boundary: this may be attributed to a variation in the transition temperature induced by local strain (Supplementary Fig. 2, Supplementary Note 2).

### Charge-order domain walls in single crystalline region

**Figure 2:**
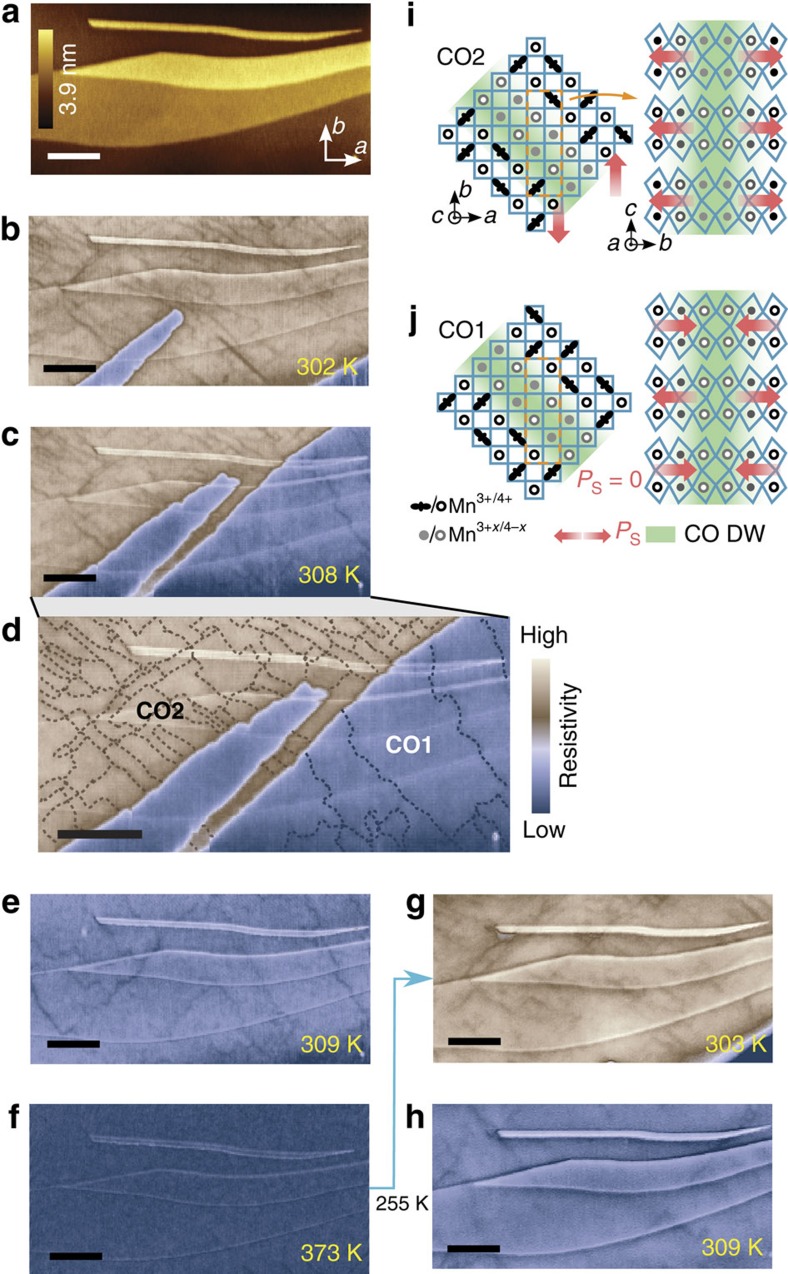
Charge-order domain walls with enhanced conductivity observed by MIM. (**a**) Surface topography of the single crystalline region, with step edges corresponding to single bilayers. (**b**–**f**) High-contrast MIM-Re images taken during warm up from 255 K, showing fine linear features, interpreted as charge-order domain walls, in addition to the CO2/CO1 phase transition. The features in **c** are highlighted in **d**. A two-tone nonlinear colour scale is used to represent the two bulk phases: darker represents a lower resistivity within both tones. Curves corresponding to step edges are used as landmarks. (**g**,**h**) MIM-Re images of the same areas after a complete thermal cycle. (**i**,**j**) In-plane (*a–b* plane) and cross-section (*b–c* plane) illustration of the charge-order domain walls in the ferroelectric CO2 (**i**) and anti-ferroelectric CO1 phase (**j**), showing also the in-plane polarization. Atoms in the first bilayer of the cross-section correspond to those enclosed by the orange dashed box in-plane. The full colour scale corresponds to 0.35 V in **b**–**f** and 0.45 V in **g**,**h**. Scale bars, 3 μm.

In a twin-free single crystalline region, linear features with enhanced conductivity in both CO1 and CO2 phases were observed in MIM-Re images (Fig. 2). The entire region is atomically flat with only a few ∼1 nm step edges corresponding to single bilayers (Fig. 2a). To minimize the effect of hysteresis at the CO1–CO2 phase transition^20^, we first cool the sample to 255 K and then collect temperature-dependent MIM images from 302 to 373 K (Fig. 2b–f). At 302 K most of the scan area is in CO2 phase (yellow–brown), which gradually shrinks until being completely replaced by CO1 phase (blue) at 309 K. Aside from the major contrast between CO2 and CO1 phases, fine linear features are clearly visible within each CO phase. The linear features have an apparent width similar to the MIM spatial resolution of ∼200 nm in this experiment, show a preferential orientation along the *a*±*b* directions and are stable from one scan to the next. They appear as lines with suppressed MIM-Re signal and thus represent regions that are more conductive than the surrounding CO phase (Fig. 1b). In Fig. 2d we marked out the clearly visible features in Fig. 2c as a guide to the eye. It is clear that they mostly form closed loops with fourfold vortices (within the spatial resolution) or end at phase boundaries. They remain stable within the CO1 and CO2 phases across multiple step edges, but undergo a drastic change passing from CO1 to CO2 phase: the linear features are considerably denser in the CO2 phase. On heating towards the CO1/non-CO transition temperature of ∼370 K, the linear features gradually become fainter and disappear completely at 363 K and higher (Fig. 2f). After a thermal cycle the linear features appear qualitatively similar, but their precise configuration has changed in both phases (Fig. 2g,h).

Aside from twin boundaries and phase boundaries between CO1 and CO2 seen in Fig. 1, there can exist a third class of boundaries in this material—boundaries between charge-order domains with a *π* shift in CO pattern, which we will refer to as charge-order domain walls (CO DWs) (Fig. 2i,j). The observed properties of the conductive linear features are compatible with those expected for CO DWs. CO DWs are unlikely to be atomically sharp: the Mn^3+/4+^ will tend to deviate towards Mn^3+*x*/4−*x*^ at the DWs to reduce interfacial Coulomb energy. They therefore represent finite-width line defects that locally lift the Mn^3+/4+^ charge order, and as such will be more conductive than the bulk CO phase, since the Coulomb energy cost for *e*_*g*_ electron hopping along the DW will be substantially smaller than in the bulk of this charge-ordered insulator. Such DWs would naturally favour *a*±*b* over *a* or *b* directions, as seen in MIM images, to avoid accumulating a net valence charge and hence a Coulomb energy penalty (Fig. 2i,j, Supplementary Fig. 3). One would also expect the CO domains to differ in length scale for different CO phases and to disappear completely in the non-CO phase, again agreeing with experimental observations. The randomization after thermal cycles further rules out the features being microscopic crystalline defects, twins or topographic features. The above evidence indicates charge-order domain walls as the most plausible interpretation of our experimental observations.

Electric polarization in PSCMO originates from the charge order superposed on the orthorhombically distorted bilayer crystal structure, so charge-order domain walls are also domain walls between in-plane polarizations in each bilayer (Fig. 2i,j). However, since conductive domain walls are observed in both the ferroelectric CO2 and anti-ferroelectric CO1 phase, we can infer that their conduction is not due to ferroelectricity. While the material is too conductive for lateral piezoresponse force microscopy (Supplementary Fig. 4, Supplementary Note 3), optical second-harmonic generation microscopy has provided evidence of *a*±*b* ferroelectric/CO domain walls in the ferroelectric CO2 phase^23^. However, second-harmonic generation microscopy cannot resolve anti-ferroelectric/CO domain walls in the anti-ferroelectric CO1 phase. MIM is able to image domain walls in the CO2, as well as CO1, phase by probing their enhanced conductivity, which relies on local lifting of charge order instead of any ferroelectric properties. Charging/band bending at domain walls due to the polarization, which could occur in the CO2 phase, is not observed to have a major effect on the domain wall conductivity. If band bending effects were to dominate, one would expect tail-to-tail domain walls to have a higher conductivity and head-to-head walls to have a lower conductivity than the *p*-typed bulk^28^, which is not observed. Therefore, the enhanced conductivity at CO DWs is likely a unique emergent phenomenon in a chemically homogeneous system that does not rely on excess charge accumulation at the interface.

### Comparison with simulations

**Figure 3:**
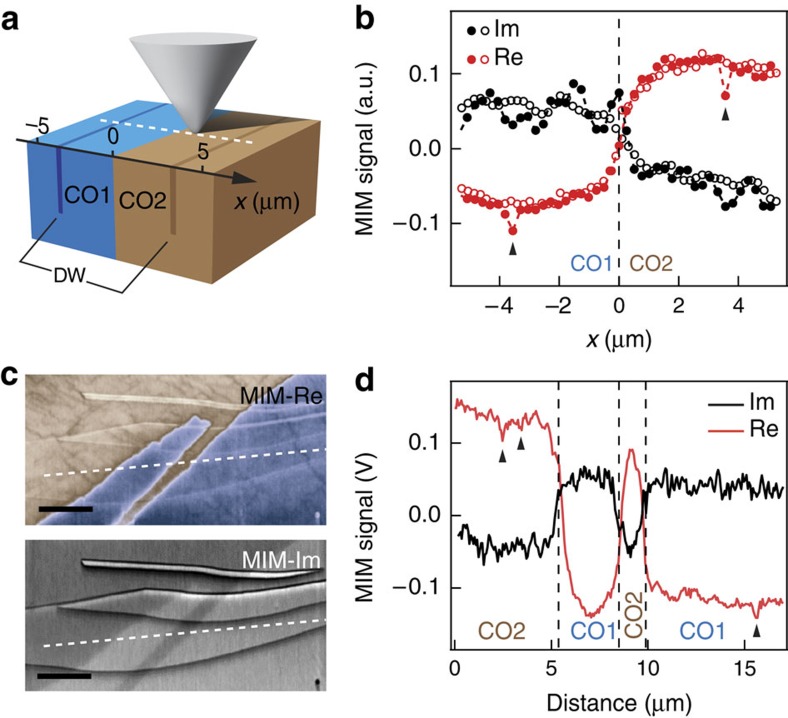
Comparison of 3D finite element analysis and experimental data of MIM response on DWs. (**a**) 3D model of the MIM setup that includes a full size tip, a large sample and two embedded 2-nm-wide DWs. The boundary between two bulk phases is at 0 μm and the two DWs are at ±3.5 μm. The in-/out-of-plane resistivity used in the simulation is (in Ω cm) 1/200 for CO1, 3/600 for CO2, 0.25/50 for both DWs. (**b**) Simulated MIM-Re and -Im trace (solid circles) when the tip is scanned across the white dashed line in **a**. Open circles are results without DWs for comparison. Clear contrast is seen between CO1/CO2 phase as well as at the DWs in MIM-Re. Due to the four orders of magnitude in length scale involved in the 3D model, numerical errors give rise to substantial noise especially in MIM-Im. (**c**) MIM images of PSCMO as in Fig. 2c, showing both MIM-Re and MIM-Im channels. Note the strong crosstalk from topographic step edges (Fig. 2a) in MIM-Im. (**d**) Line-cuts corresponding to the white dashed lines in **c**: positions of DWs are indicated. Scale bars, 3 μm.

Because MIM measures the perturbation of tip–sample admittance, features below the spatial resolution may not be detectable if the admittance perturbation that such features generate is too small. To prove that MIM working at 1 GHz has enough sensitivity to image features a few nanometres wide with reasonable conductivity contrast, we employed three-dimensional finite element analysis. The simulation includes two bulk regions to represent CO1/CO2 phase separation and narrow stripes (2 nm wide) with a conductivity higher than the bulk within each phase to represent DWs (Fig. 3). Tip–sample admittance is calculated as the tip moves across the sample: we can thus obtain simulated MIM line-cuts.

Good agreement is achieved between simulation results (Fig. 3b) and actual data (Fig. 3d). The in-plane/out-of-plane resistivity of both DWs set to be 0.25/50 Ω cm—a conservative extrapolation of the non-CO resistivity (Fig. 1a) to 300 K. The simulated MIM-Re line-cut shows clear contrast at the DWs, with a resolution of ∼300 nm determined by the tip apex size. We have thus shown that MIM has enough sensitivity to generate contrast comparable to experimental values for reasonably conductive DWs with width down to few nanometres in PSCMO. In reality, DWs may be wider (up to the spatial resolution) and less conductive (down to bulk conductivity), but as long as they cause the same perturbation to the tip–sample admittance, the resulting MIM contrast will be similar. A more quantitative characterization is therefore only possible with sharper MIM probes. Additional simulations also confirm the sensitivity of MIM to less conductive DWs with width down to a few nanometres: since these were never observed in MIM images we can conclude that all DWs are more conductive than the bulk, contrary to the band bending picture but consistent with the charge-order defect picture.

### Charge-order domain walls in the presence of structural twins

**Figure 4:**
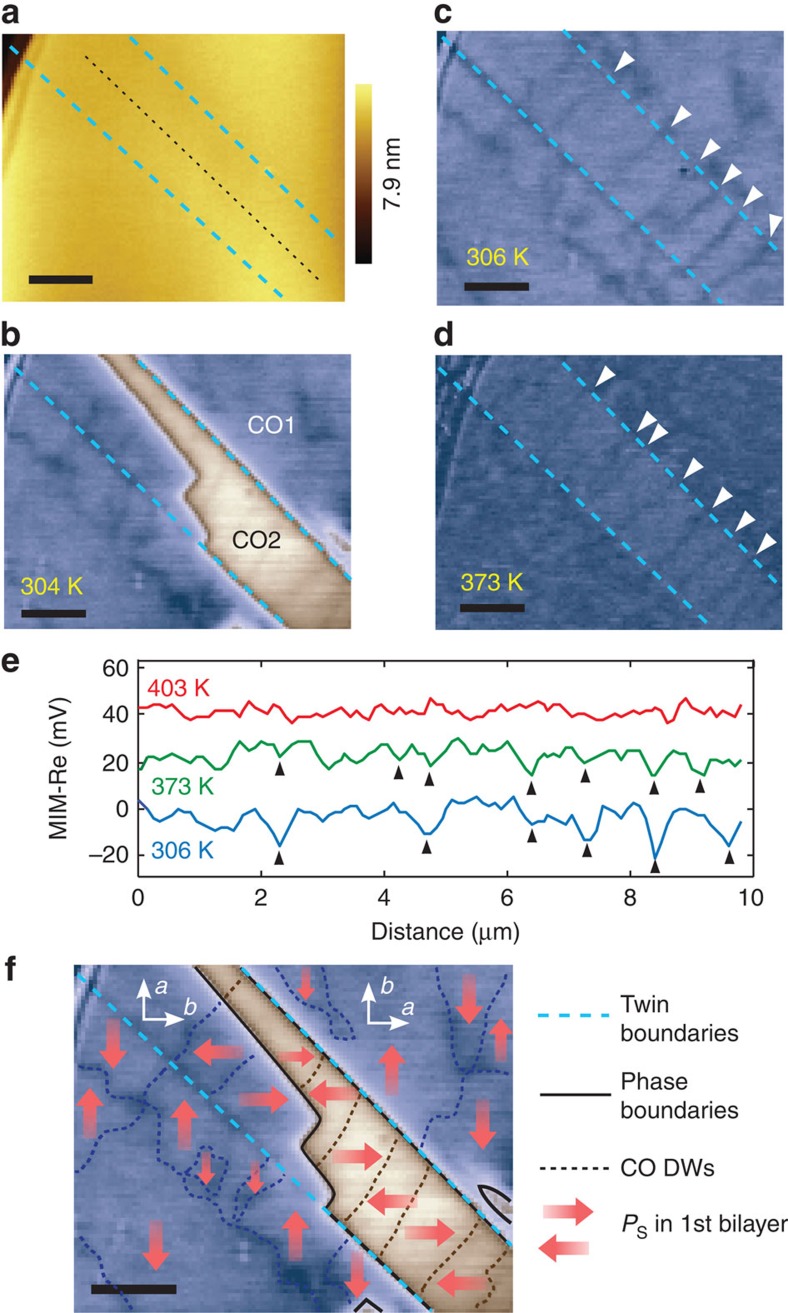
Charge-order domain walls in the presence of structural twins. (**a**) Surface topography of the twinned region. Twin boundaries are marked by cyan dashed lines. The majority of the region is atomically flat. (**b**–**d**) High contrast MIM-Re images showing charge-order domain walls. The DWs in the narrow twinned region are indicated by white arrowheads. The presence of structural twin imposes a strong preference of domain wall orientation and stabilizes the domains at up to 373 K. (**e**) Three-pixel averaged line-cuts of the MIM-Re images along the dotted black line in **a**, at various temperatures. Most DWs remain unchanged from 306 (blue) to 373 K (green) with a few exceptions, until disappearing at 403 K (red). Traces at 373 and 403 K are offset for visibility. (**f**) Illustration of three-hierarchy twin/phase/CO domain boundaries based on **b**. The absolute polarization direction can differ from that illustrated by a *π* rotation. Microscopic domains below the spatial resolution may also occur. Scale bars, 2 μm.

Detailed MIM scans of the twinned region in Fig. 1 reveal that CO DWs are strongly affected by the presence of structural twins (Fig. 4). The most distinct feature is the uniformly oriented, quasi-periodically spaced DWs inside the narrow twinned region (Fig. 4c,d). Remarkably, most DWs inside remain stable and clearly visible at up to 373 K, at which temperature the domain structure outside has melted completely (Fig. 4d, green trace in Fig. 4e). Further heating to 403 K is required to melt all CO in the entire sample (Fig. 4e red trace). This implies that the domain structure and underlying CO1 phase are stabilized by structural twins. These phenomena are likely due to the combined effect of highly anisotropic boundary condition and strains: the twinned region is much longer in one direction than the other, making closely spaced DWs perpendicular to the long axis more energetically favourable and stable in the presence of strains, as reported in other systems under similar conditions^29^,^30^. For use in nanoscale devices^31^,^32^, the effect of strain and geometry may be exploited to reliably create domain walls with controlled orientation and quantity.

### History dependence of charge-order domains in CO1 phase

**Figure 5:**
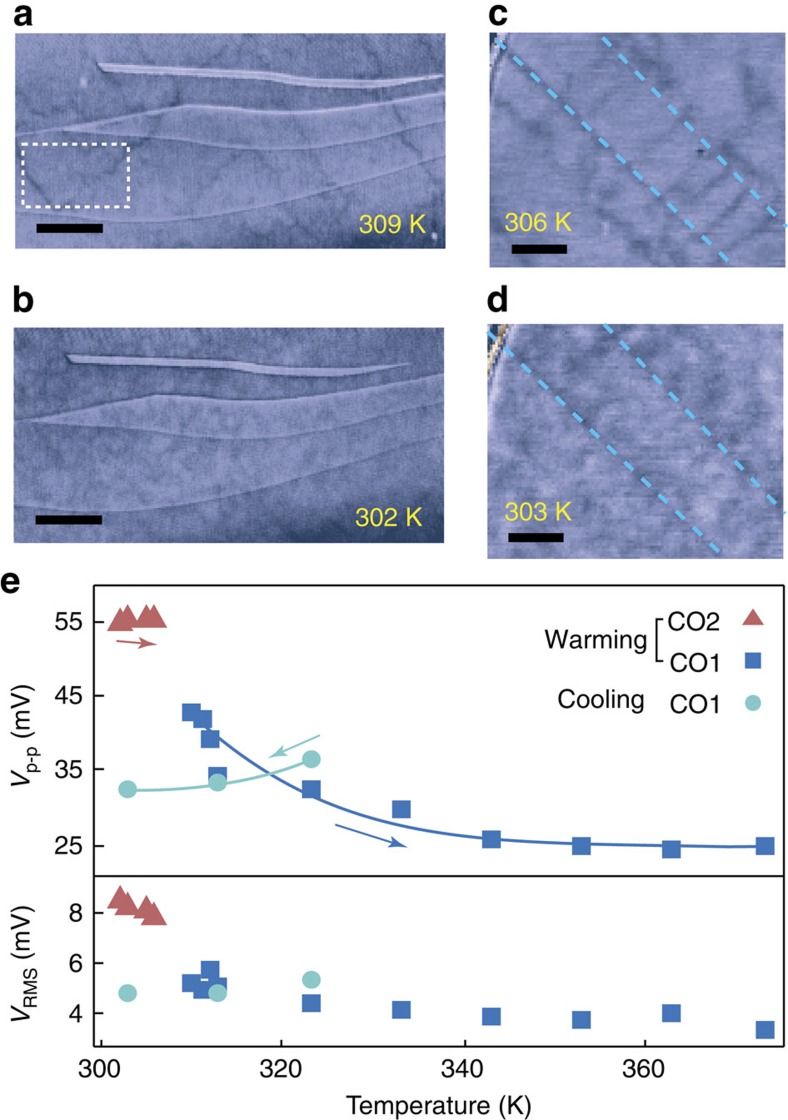
Thermal history dependence of CO domains in the CO1 phase. (**a**,**c**) MIM-Re images of CO domains warming up from 255 K for single crystalline (**a**) and twinned region (**c**), showing large, well-defined domains. (**b**,**d**) The same regions after cooling down to room temperature from 373 and 403 K, respectively, showing drastically different patterns. (**e**) Thermal history dependence of the peak-to-peak and RMS values of the MIM-Re signal inside the white dotted box in **a**. Scale bars, 3 μm in **a**,**b** and 2 μm in **c**,**d**.

Thermal cycles reveal that the CO DWs have a strong history dependence. Because the CO1–CO2 transition is hysteretic (Fig. 1a), to ensure consistent sample preparation we usually perform complete thermal cycles by cooling the sample to 255 K after reaching the high-temperature non-CO phase. Interestingly, a different domain structure in the CO1 phase is seen when cooling from the non-CO phase (≥373 K) than when warming from the CO2 phase (255 K). Large (≤2 μm), regular domains as seen after warming from 255 to 309 K (Fig. 5a,c) were not observed after cooling from ≥373 K (Fig. 5b,d), even though in both cases the phase is the same (CO1). Instead, the MIM signal varies over a much smaller length scale but with a similar magnitude as can be seen from peak-to-peak and root mean square (RMS) values (Fig. 5e). The phenomenon is robust for average warming and cooling rates ranging from ∼0.2 to 5 K min^−1^ and shows negligible time dependence while staying at room temperature for 7 h. Large domains are observed to re-appear after cooling to 255 K and warming up again. We may infer that the CO domains have been reduced in size when cooling from the non-CO phase, becoming too small for MIM to clearly distinguish, possibly due to higher nucleation centre density. This thermal history dependence may be exploited to manipulate domain/domain wall density in nanoscale devices by local Joule heating^33^.

## Discussion

We have completely mapped the mesoscale surface topography, crystalline orientation and electronic phases in a bilayer PSCMO crystal by combining PLM with MIM. Conductive features are observed in both charge-ordered phases, and are identified as charge-order domain walls. The domain walls obtain enhanced conductivity as line defects in the charge order, show strong history dependence and are stabilized by the presence of structural twins. MIM measurements allow us to map the full hierarchy of phase boundaries in a bulk complex oxide crystal—structural twins, CO1/CO2 phase boundaries, and CO DWs—in a single image (Fig. 4f). Our findings suggest numerous interesting possibilities of exploiting this new class of filamentary conduction, by local strain, heating, electric field or anisotropic geometry, to create nanoscale electronic or memory devices^31^,^32^,^33^.

## Methods

### Sample preparation

Single-crystal samples of PSCMO were grown by a floating zone method. Crystals were cleaved in air to expose a clean *a−b* plane surface for optical microscopy and MIM. Elongated structural twins of width ranging from hundreds of microns to hundreds of nanometres are present. The structural twins are roughly along the *a*±*b* direction with straight boundaries, as commonly seen in this material^19^.

### MIM measurement and response curve calculation

MIM imaging was carried out using an atomic force microscopy-based system, operated in air at room temperature and higher, in constant-force contact mode. Images proportional to (up to a constant shift) the real (resistive) and imaginary (capacitive) parts of the complex tip–sample admittance at 1 GHz are referred to as MIM-Re and MIM-Im^26^. The MIM response curve in Fig. 1b is simulated using finite element analysis in COMSOL Multiphysics 4.4 (COMSOL, Inc., Palo Alto, CA 94304, USA, Supplementary Fig. 1) for a layered material with out-of-plane resistivity equal to 200 times the average in-plane resistivity. The real dielectric constant (*ɛ*′) is set to be 20 and does not have a major effect on the MIM response. The imaginary dielectric constant (*ɛ*′′, or dielectric loss) is irrelevant due to the high conductivity of the material. Because of the long-range nature of microwave coupling and the fact that the MIM tip is not shielded to the apex, the response curve in Fig. 1b cannot be used quantitatively on small features because the sample was assumed to be very large in the simulation; three-dimensional simulation is needed in this case as described in the main text.

## Additional information

**How to cite this article:** Ma, E. Y. *et al.* Charge-order domain walls with enhanced conductivity in a layered manganite. *Nat. Commun.* 6:7595 doi: 10.1038/ncomms8595 (2015).

## Supplementary Material

Supplementary Information Supplementary Figures 1-4, Supplementary Notes 1-3 and Supplementary References

## References

[b1] Ohtomo A. & Hwang H. Y. A high-mobility electron gas at the LaAlO_3_/SrTiO_3_ heterointerface. Nature 427, 423–427 (2004). 14749825 10.1038/nature02308

[b2] Lee J. J. *et al.* Interfacial mode coupling as the origin of the enhancement of Tc in FeSe films on SrTiO_3_. Nature 515, 245–248 (2014). 25391962 10.1038/nature13894

[b3] Goncalves-Ferreira L., Redfern S., Artacho E. & Salje E. Ferrielectric twin walls in CaTiO_3_. Phys. Rev. Lett. 101, 097602 (2008). 18851659 10.1103/PhysRevLett.101.097602

[b4] Garcia V. *et al.* Ferroelectric control of spin polarization. Science 327, 1106–1110 (2010). 20075211 10.1126/science.1184028

[b5] Van Aert S. *et al.* Direct observation of ferrielectricity at ferroelastic domain boundaries in CaTiO_3_ by electron microscopy. Adv. Mater. 24, 523–527 (2012). 22223264 10.1002/adma.201103717

[b6] Fernandes Vaz C. A. & Staub U. Artificial multiferroic heterostructures. J. Mater. Chem. C 1, 6731–6742 (2013).

[b7] Seidel J. *et al.* Conduction at domain walls in oxide multiferroics. Nat. Mater. 8, 229–234 (2009). 19169247 10.1038/nmat2373

[b8] Choi T., Lee S., Choi Y. J., Kiryukhin V. & Cheong S.-W. Switchable ferroelectric diode and photovoltaic effect in BiFeO_3_. Science 324, 63–66 (2009). 19228998 10.1126/science.1168636

[b9] Wu W., Horibe Y., Lee N., Cheong S.-W. & Guest J. R. Conduction of topologically protected charged ferroelectric domain walls. Phys. Rev. Lett. 108, 077203 (2012). 22401247 10.1103/PhysRevLett.108.077203

[b10] Schröder M. *et al.* Conducting domain walls in lithium niobate single crystals. Adv. Funct. Mater. 22, 3936–3944 (2012).

[b11] Maksymovych P. *et al.* Tunable metallic conductance in ferroelectric nanodomains. Nano Lett. 12, 209–213 (2012). 22181709 10.1021/nl203349b

[b12] Vasudevan R. K. *et al.* Domain wall geometry controls conduction in ferroelectrics. Nano Lett. 12, 5524–5531 (2012). 22994244 10.1021/nl302382k

[b13] Morozovska A. N., Vasudevan R. K., Maksymovych P., Kalinin S. V. & Eliseev E. A. Anisotropic conductivity of uncharged domain walls in BiFeO_3_. Phys. Rev. B 86, 085315 (2012).

[b14] Vasudevan R. K. *et al.* Domain wall conduction and polarization-mediated transport in ferroelectrics. Adv. Funct. Mater. 23, 2592–2616 (2013).

[b15] Sluka T., Tagantsev A. K., Bednyakov P. & Setter N. Free-electron gas at charged domain walls in insulating BaTiO_3_. Nat. Commun. 4, 1808 (2013). 23651996 10.1038/ncomms2839 PMC3674246

[b16] Renner C., Aeppli G., Kim B.-G., Soh Y.-A. & Cheong S.-W. Atomic-scale images of charge ordering in a mixed-valence manganite. Nature 416, 518–521 (2002). 11932740 10.1038/416518a

[b17] Tokura Y. Critical features of colossal magnetoresistive manganites. Reports Prog. Phys. 69, 797–851 (2006).

[b18] Li Q. *et al.* Prediction and experimental evidence for thermodynamically stable charged orbital domain walls. Phys. Rev. X 4, 031028 (2014).

[b19] Tokunaga Y. *et al.* Rotation of orbital stripes and the consequent charge-polarized state in bilayer manganites. Nat. Mater. 5, 937–941 (2006). 17086170 10.1038/nmat1773

[b20] He Z. B., Deng G., Tian H., Xu Q. & Van Tendeloo G. 90° Rotation of orbital stripes in bilayer manganite PrCa_2_Mn_2_O_7_ studied by in situ transmission electron microscopy. J. Solid State Chem. 200, 287–293 (2013).

[b21] He Z., Tian H., Deng G., Xu Q. & Van Tendeloo G. Microstructure of bilayer manganite PrCa_2_Mn_2_O_7_ showing charge/orbital ordering. Appl. Phys. Lett. 102, 212902 (2013).

[b22] Yamauchi K. & Picozzi S. Mechanism of ferroelectricity in half-doped manganites with pseudocubic and bilayer structure. J. Phys. Soc. Japan 82, 1–5 (2013).

[b23] Itoh H., Tokunaga Y., Kida N., Shimano R. & Tokura Y. Charge-ordering-induced polar domains and domain walls in a bilayered manganite Pr(Sr_0.15_Ca_0.85_)_2_Mn_2_O_7_. Appl. Phys. Lett. 96, 032902 (2010).

[b24] Kundhikanjana W., Lai K., Kelly M. A. & Shen Z.-X. X. Cryogenic microwave imaging of metal-insulator transition in doped silicon. Rev. Sci. Instrum. 82, 033705 (2011). 21456749 10.1063/1.3554438

[b25] Lai K. *et al.* Mesoscopic percolating resistance network in a strained manganite thin film. Science 329, 190–193 (2010). 20616272 10.1126/science.1189925

[b26] Kundhikanjana W. *et al.* Hierarchy of electronic properties of chemically derived and pristine graphene probed by microwave imaging. Nano Lett. 9, 3762–3765 (2009). 19678669 10.1021/nl901949z

[b27] Lee Y., Tokunaga Y., Arima T. & Tokura Y. Change in optical anisotropy with rotation of orbital stripe in a half-doped bilayer manganite. Phys. Rev. B 75, 174406 (2007).

[b28] Meier D. *et al.* Anisotropic conductance at improper ferroelectric domain walls. Nat. Mater. 11, 284–288 (2012). 22367003 10.1038/nmat3249

[b29] Seul M. & Andelman D. Domain shapes and patterns: the phenomenology of modulated phases. Science 267, 476–483 (1995). 17788780 10.1126/science.267.5197.476

[b30] Wei J., Wang Z., Chen W. & Cobden D. New aspects of the metal–insulator transition in single-domain vanadium dioxide nanobeams. Nat. Nanotechnol. 4, 420–424 (2009). 19581893 10.1038/nnano.2009.141

[b31] Hehn M. *et al.* Nanoscale magnetic domains in mesoscopic magnets. Science 272, 1782–1785 (1996). 8662483 10.1126/science.272.5269.1782

[b32] Cao J. *et al.* Strain engineering and one-dimensional organization of metal-insulator domains in single-crystal vanadium dioxide beams. Nat. Nanotechnol. 4, 732–737 (2009). 19893528 10.1038/nnano.2009.266

[b33] Hamann H. F., O'Boyle M., Martin Y. C., Rooks M. & Wickramasinghe H. K. Ultra-high-density phase-change storage and memory. Nat. Mater. 5, 383–387 (2006). 16604077 10.1038/nmat1627

