# Supplementary material for: Charge-order domain walls with enhanced conductivity in a layered manganite
Source: Nat Commun. 2015 Jul 3;6:7595. doi: 10.1038/ncomms8595 (PMC4506533; doi:10.1038/ncomms8595)
Supplement: Supplementary Information — Supplementary Figures 1-4, Supplementary Notes 1-3 and Supplementary References [file ncomms8595-s1.pdf]

# Supplementary Figures

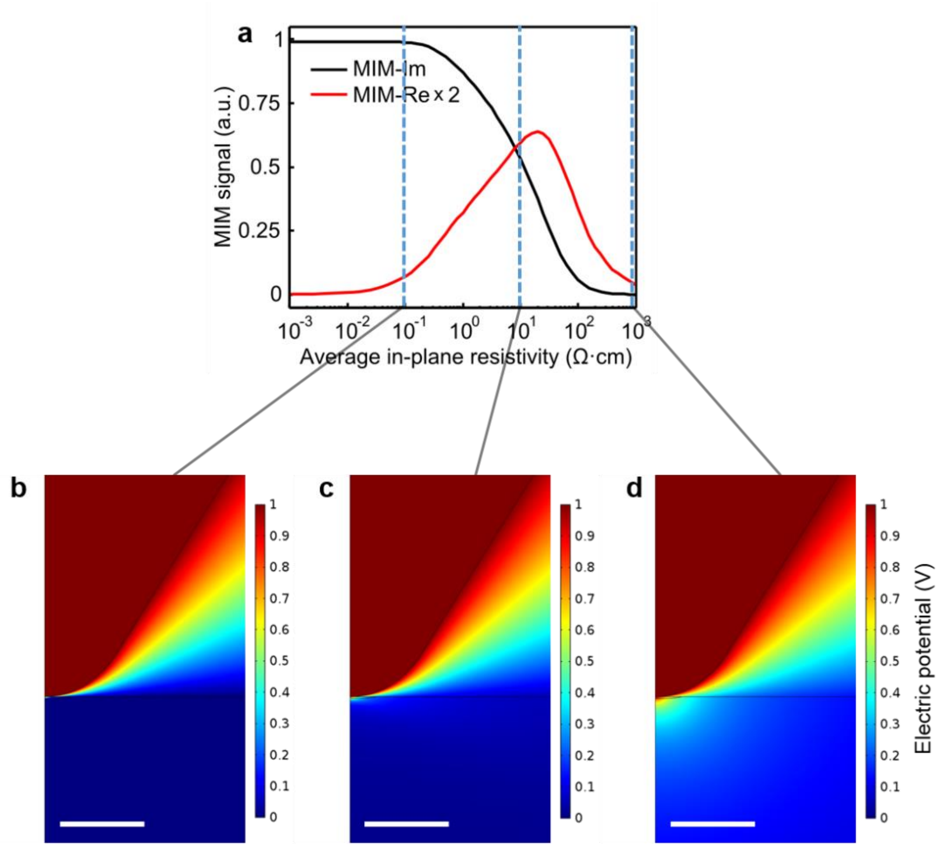


**Supplementary Figure 1. 2D axisymmetric finite element analysis for MIM response curve on PSCMO. (a)** MIM response as a function of average in-plane resistivity of the sample, as in figure 1b. **(b)-(d)** Zoomed in view of the 2D model showing electric potential distribution near the tip apex at various values of sample resistivity. The potential of the tip is 1 V. The field is mostly concentrated within a few radii under the tip apex, confirming the near field nature of MIM. However since the tip is not shielded all the way to the apex, the contribution of the upper part of the cone to the total tip-sample impedance is non-negligible: this results in the quantitative value of MIM contrast being smaller for smaller features than for large features with the same resistivity difference. In practice this means the MIM response curve can only be regarded as a qualitative measure of local resistivity. The scale bars are 100 nm. More details see Supplementary Note 1.

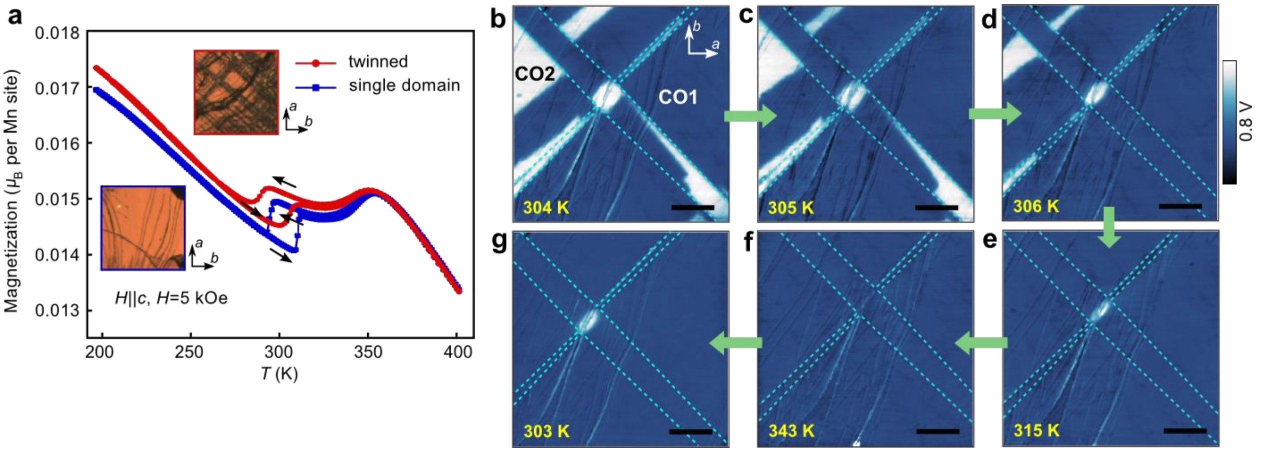


**Supplementary Figure 2. Real space imaging of nucleation during the CO1-CO2 phase transition. (a)** Magnetization measurement of two samples with very different populations of structural twins, as shown by the inset cross-polarized light microscope images. The phase transition in the heavily twinned sample is broadened. **(b)-(f)** MIM-Re images of the same area as in Fig. 1 during a continuous heating from 304 to 343 K, after cooling to 255 K. The structural twin boundaries are marked by cyan dashed lines. Nucleation of the CO2 phase by twin boundaries may be observed. **(g)** Same area after cooling down from 403 K (not shown), showing delayed emergence of CO2 phase. The scale bars are 5 µm. See also Supplementary Note 2.

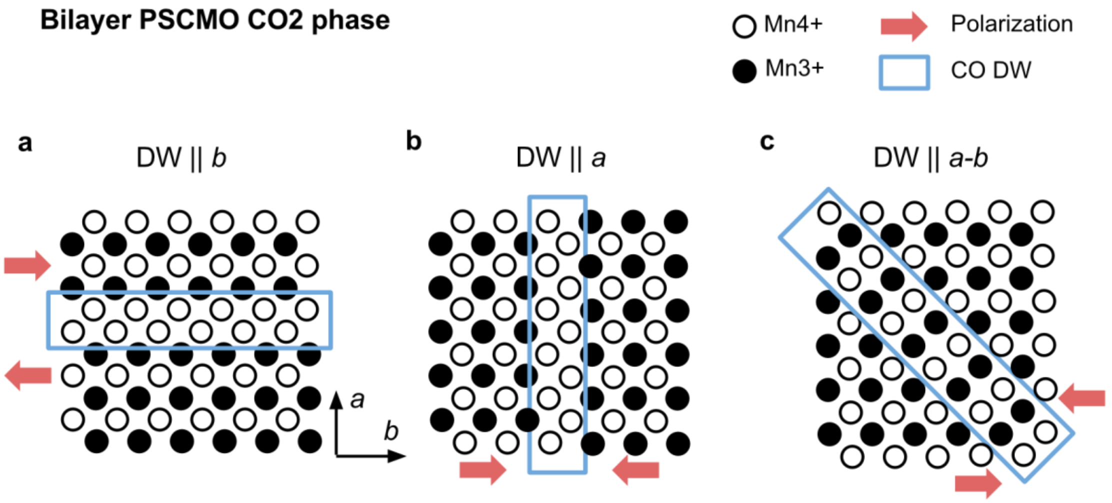


**Supplementary Figure 3. Illustration of DWs along different crystal directions: (a)** ||*a*, **(b)** ||*b* and **(c)** ||*a*-*b*. Coulomb energy penalty from ordered charge is drastically reduced for *a*±*b* DWs by avoiding net charge accumulation. The contribution from ferroelectricity is much smaller in comparison and thus is not expected to determine the preferred DW orientation.

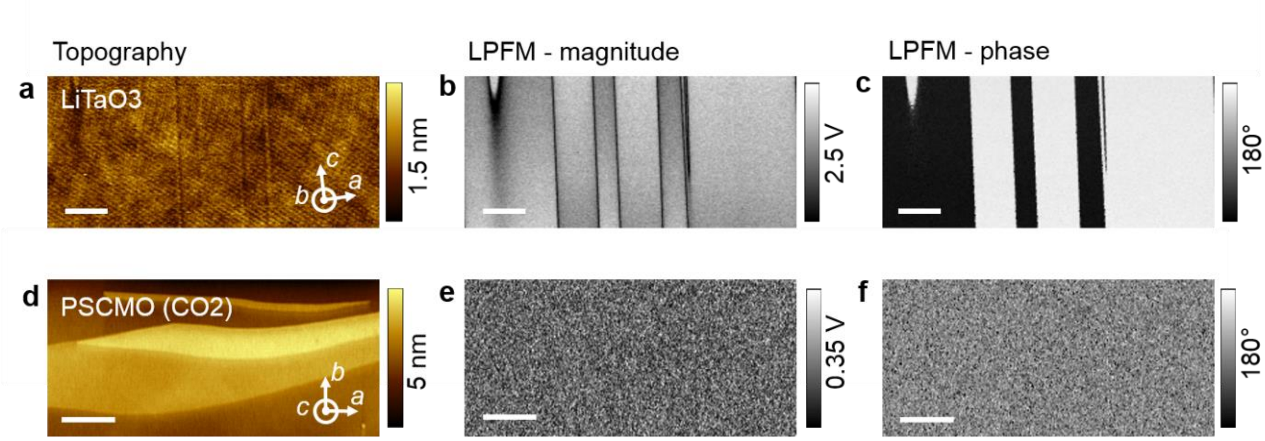


**Supplementary Figure 4. Lateral piezoresponse force microscopy (LPFM) results on $LiTaO_3$ and bilayer PSCMO. (a)-(c)** Topography, LPFM-magnitude and phase images of a $LiTaO_3$ sample. Polarizations are along ±c direction. Clear phase contrast is seen between in-plane ferroelectric domains. **(d)-(f)** Same measurement yielded featureless images for the bilayer PSCMO sample due to its high conductivity. The excitation is 1 V@5 kHz for $LiTaO_3$ and 2 V@5 kHz for PSCMO. The scale bars are 5 µm for $LiTaO_3$ and 3 µm for PSCMO. See Supplementary Note 3 for the discussion.

# Supplementary Notes

## Supplementary Note 1. Calculation of MIM response curves

The MIM response curves in main text Fig. 1b are obtained by finite element analysis, using COMSOL Multiphysics 4.4. The signals in MIM-Im(Re) channel are linearly proportional to the imaginary(real) part of the complex tip-sample admittance at 1 GHz. Because the relevant length scale of tip-sample interaction (<10 μm) is much smaller than the wavelength of electromagnetic wave at 1 GHz (~30 cm in air, ~20 cm in the waveguides used in MIM), this admittance is easily obtained with a quasi-static model.

A 2D axisymmetric model is used (Supplementary Fig. 1). The sample is set to be homogeneous and effectively infinite for the tip. The tip is modelled as a cone with an apex radius of 100 nm. The cone angle is obtained from SEM images of the tip. For simplicity, the bilayer PSCMO is modelled as an anisotropic material with an out-of-plane resistivity 200 times the value of the average in-plane resistivity, as obtained from the transport measurements (Fig. 1a). There is a 2 nm thick insulating depletion layer right under the contact point to account for the non-ohmic contact[1]. A real dielectric constant ($\varepsilon$') of 20 is used. The imaginary dielectric constant ($\varepsilon$'', or dielectric loss) is irrelevant due to the high conductivity of the material: $\varepsilon$'' would need to be >~3000 to be comparable to the contribution from conduction electrons even in the most insulating phase.

Average in-plane resistivity (and out-of-plane resistivity accordingly) is varied as a parameter and the imaginary(real) part of the admittance between the tip and the grounding plane below the sample is calculated from the simulated field distribution (Supplementary Fig. 1b-d) and plotted as the MIM-Im(Re) response curve. The asymmetric shape, different from that for isotropic materials[2], is due to the anisotropy in resistivity. Varying the depletion layer thickness between 1-10 nm or the real dielectric constant between 5-100 does not change the response curves qualitatively in the relevant resistivity regime.

The ability to respond to local conductivity without the need of a nearby electrode – thus removing the complication of current paths and non-Ohmic contact effects – make MIM a practical alternative to conductive atomic force microscopy (cAFM) in studying bulk crystal samples. We tried putting back electrodes on several thin crystals (<~200 μm) and scanning in cAFM mode with a commercial Pt/Ir coated probe (Applied NanoStructures Inc.), but were not able to get a stable contact even with a bias of up to ±10 V, which ultimately burned the contact area. Moreover, the GHz frequency used is high enough

to enable MIM to sense subtle conductivity differences even in fairly conductive materials (<~1 Ω·cm) which would be difficult for techniques using capacitive coupling at lower frequencies, for example electrostatic force microscopy (EFM)[3].

## Supplementary Note 2. Nucleation during the CO1-CO2 phase transition

In a strongly correlated complex oxide like the bilayer PSCMO studied here, strain can substantially affect transition temperatures[4]; localized strain, for example induced by grain boundaries, may produce a substantial microscopic variation in ordering temperatures in oxides[5]. Supplementary Fig. 2a shows a bulk magnetization measurement of two macroscopic samples of PSCMO with very different amounts of twinning: a broader phase transition is observed in the twinned sample than in a single-domain sample.

Since by using MIM we are able to unambiguously distinguish the two charge order phases, we can visualize phase coexistence during the CO1-CO2 phase transition and demonstrate the variation in transition temperature, due to local strain, at the microscopic level. Supplementary Fig. 2b-f show temperature dependent MIM-Re images of the same area with moderate twinning as in Fig. 1 up to 343 K. As the sample is warmed up, the CO2 phase gradually disappears, but survives to higher temperature near structural twin boundaries, in stark contrast with the rapid phase transition in the single crystal region (main text Fig. 2). This effect may be attributed to the large variation in local strain due to the twin boundaries.

The CO2 phase disappears completely at 343 K; we heat the sample further to 403 K as in the magnetization measurement. During the subsequent cooling process, the CO2 phase only starts to appear when reaching 303 K (Supplementary Fig. 2g), consistent with the hysteresis observed in bulk measurements.

## Supplementary Note 3. Effects of the charge order domain walls being also anti-ferro/ferroelectric domain walls

In both CO1 and CO2 phases, spontaneous polarization ($P_s$) in the $\pm b$ direction emerges due to the "checkerboard" CO pattern superposed on the orthorhombically distorted lattice in which Mn-O-Mn bond lengths and angles alternate with the same periodicity as that of the CO[6]. The CO DWs, separating CO

domains with a π shift in CO pattern but a continuous lattice, are thus also ferroelectric domain walls. The difference between the two CO phases is that polarizations in adjacent bi-layers are in phase in the CO2 phase (ferroelectric) but out of phase in the CO1 phase (layered anti-ferroelectric) (main text Fig. 2i, j). With a sizable polarization of ~1.8 $\mu C/cm^2$,[6] one in principle could study the domain structure in the CO2 phase with lateral piezoresponse force microscopy (LPFM)[7] but this has proven extremely difficult due to the substantial leakage in the material (Supplementary Fig. 4).

Supplementary Fig. 4a-c show the lateral piezoresponse force microscopy (LPFM) results on a $LiTaO_3$ test sample with in-plane ferroelectric domains. The material is insulating with a polarization of ~50 $\mu C/cm^2$ [8]and we were able to resolve the domains with excellent signal-noise ratio (Supplementary Fig. 4c). We then attempted to study the bilayer PSCMO in the ferroelectric CO2 phase but did not get any contrast (Supplementary Fig. 4e, f) – the polarization in CO2 phase is ~1.8 $\mu C/cm^2$,[6] which should be readily detectable if the material were insulating enough.

Previous experiments on PSCMO using second-harmonic generation microscopy provided evidence of ferroelectric domain walls along the $a \pm b$ directions, but only in the ferroelectric CO2 phase, not the layered anti-ferroelectric CO1 phase[9]. This is because the polarization averages to zero along c direction in the CO1 phase (Fig. 2j). In contrast, MIM is able to resolve the anti-ferro/ferroelectric domain walls due to their enhanced conductivity, which arises from the local suppression of charge order. This property applies to both “head-to-head (HH)” and “tail-to-tail (TT)” DWs, in both CO1 and CO2 phases, so all domain walls show an enhanced conductivity. Band bending induced by excess charge[10,11] which was used to explain ferroelectric DW conductivity in several other ferroelectric materials without charge ordering, is not expected to play a major role in this material: if band bending effects were to dominate, in this *p*-type material[12] one would expect tail-to-tail domain walls to have a higher conductivity while head-to-head walls to have a lower conductivity than the bulk[13]; while less conductive DWs are readily detectable by MIM, they were never observed. Moreover, because the contribution of band bending to conductivity would be destructive between adjacent bi-layers in the anti-ferroelectric CO1 phase, most of the effect would be neutralized and the DW conductivity would be substantially different depending on whether the DW in the first exposed bilayer is HH or TT type; nonetheless we see clear enhanced DW conductivity that remains stable across multiple step edges of single bi-layers in CO1 phase (Fig. 2e).

## Supplementary References


1. Wu, W. *et al.* Polarization-modulated rectification at ferroelectric surfaces. *Phys. Rev. Lett.* **104,** (2010).

2. Kundhikanjana, W., Lai, K., Kelly, M. A. & Shen, Z.-X. X. Cryogenic microwave imaging of metal-insulator transition in doped silicon. *Rev. Sci. Instrum.* **82,** 033705 (2011).

3. Gupta, S., Williams, O. a. & Bohannan, E. Electrostatic force microscopy studies of boron-doped diamond films. *J. Mater. Res.* **22,** 3014–3028 (2011).

4. Tokunaga, Y. *et al.* Effects of uniaxial stress on orbital stripe direction in half-doped layered manganites: Eu0.5Ca1.5MnO4 and Pr(Sr,Ca)2Mn2O7. *Phys. Rev. B* **78,** 155105 (2008).

5. Soh, Y.-A., Aeppli, G., Mathur, N. & Blamire, M. Mesoscale magnetism at the grain boundaries in colossal magnetoresistive films. *Phys. Rev. B* **63,** 020402 (2000).

6. Yamauchi, K. & Picozzi, S. Mechanism of Ferroelectricity in Half-Doped Manganites with Pseudocubic and Bilayer Structure. *J. Phys. Soc. Japan* **82,** 1–5 (2013).

7. Roelofs, a. *et al.* Differentiating 180° and 90° switching of ferroelectric domains with three-dimensional piezoresponse force microscopy. *Appl. Phys. Lett.* **77,** 3444 (2000).

8. Gopalan, V. & Gupta, M. C. Observation of internal field in LiTaO 3 single crystals : Its origin and time-temperature dependence. *Appl. Phys. Lett.* **68,** 1995–1997 (1996).

9. Itoh, H., Tokunaga, Y., Kida, N., Shimano, R. & Tokura, Y. Charge-ordering-induced polar domains and domain walls in a bilayered manganite Pr(Sr0.15Ca0.85)2Mn2O7. *Appl. Phys. Lett.* **96,** 032902 (2010).

10. Wu, W., Horibe, Y., Lee, N., Cheong, S.-W. & Guest, J. R. Conduction of Topologically Protected Charged Ferroelectric Domain Walls. *Phys. Rev. Lett.* **108,** 077203 (2012).

11. Eliseev, E. A., Morozovska, A. N., Svechnikov, G. S., Gopalan, V. & Shur, V. Y. Static conductivity of charged domain walls in uniaxial ferroelectric semiconductors. *Phys. Rev. B - Condens. Matter Mater. Phys.* **83,** (2011).

12. Subba Rao, G. V., Wanklyn, B. M. & Rao, C. N. R. Electrical transport in rare earth ortho-chromites, -manganites and -ferrites. *Journal of Physics and Chemistry of Solids* **32,** 345–358 (1971).

13. Meier, D. *et al.* Anisotropic conductance at improper ferroelectric domain walls. *Nat. Mater.* **11,** 284–8 (2012).
